# Contrast Enhancement Algorithm Based on Gap Adjustment for Histogram Equalization

**DOI:** 10.3390/s16060936

**Published:** 2016-06-22

**Authors:** Chung-Cheng Chiu, Chih-Chung Ting

**Affiliations:** 1 Department of Electrical and Electronic Engineering, Chung Cheng Institute of Technology, National Defense University, Taoyuan 33551, Taiwan; 2 School of Defense Science, Chung Cheng Institute of Technology, National Defense University, Taoyuan 33551, Taiwan; chihchungting@gmail.com

**Keywords:** cumulative distribution function (CDF), contrast enhancement, histogram equalization (HE), human visual perception, gap adjustment

## Abstract

Image enhancement methods have been widely used to improve the visual effects of images. Owing to its simplicity and effectiveness histogram equalization (HE) is one of the methods used for enhancing image contrast. However, HE may result in over-enhancement and feature loss problems that lead to unnatural look and loss of details in the processed images. Researchers have proposed various HE-based methods to solve the over-enhancement problem; however, they have largely ignored the feature loss problem. Therefore, a contrast enhancement algorithm based on gap adjustment for histogram equalization (CegaHE) is proposed. It refers to a visual contrast enhancement algorithm based on histogram equalization (VCEA), which generates visually pleasing enhanced images, and improves the enhancement effects of VCEA. CegaHE adjusts the gaps between two gray values based on the adjustment equation, which takes the properties of human visual perception into consideration, to solve the over-enhancement problem. Besides, it also alleviates the feature loss problem and further enhances the textures in the dark regions of the images to improve the quality of the processed images for human visual perception. Experimental results demonstrate that CegaHE is a reliable method for contrast enhancement and that it significantly outperforms VCEA and other methods.

## 1. Introduction

Good lighting conditions and high sensitivity of CCD (Charge-Coupled Device)/CMOS (Complementary Metal-Oxide Semiconductor) sensors are important factors in obtaining good image quality. Strong light on the subject leads to a white or washed out appearance; conversely, weak light on the subject results in the image appearing too dark to be seen clearly. Further, poor sensitivity of CCD/CMOS sensors causes images to have obscure details and lower contrast with narrow dynamic ranges. However, image enhancement methods can effectively improve the visual quality of images and compensate the disadvantages arising from the inferior light sensitivity of CCD/CMOS sensors. Therefore, these techniques have been widely used in many fields such as image processing, medical analyses, meteorological images, consumer electronics, and other applications in recent years. They are also utilized for obtaining clearer images on the displays of various types of instruments.

Histogram equalization (HE) [1] is a simple, effective, and widely utilized image enhancement method. After performing HE, the intensity histogram of a poor contrast image spans the full spectrum of the gray levels to gain higher contrast and achieve image enhancement. Although HE enhances an image effectively, it may cause the gray values of the enhanced image to be overstretched and lead to excessive contrast enhancement. Further, HE may also result in the feature loss problem because a lot of gray values with smaller probabilities are merged to a certain gray value.

Many studies have focused on finding solutions to the over-enhancement problem produced by HE. Brightness preserving bi-histogram equalization (BBHE) [2] segments an original image histogram into two subhistograms based on its mean and performs HE in each of them. Dynamic histogram equalization (DHE) [3] segments the histogram into several subhistograms by using the local minima. When the subhistogram is not a normal distribution, DHE further partitions it into three parts by using *μ + σ* and *μ* − *σ.* Here, the values *μ* and *σ*, they denote the mean and the standard deviation of the subhistogram, individually. Then, every subhistogram is reassigned its dynamic range and HE is performed in each of them. Dynamic range separate histogram equalization (DRSHE) [4] utilizes the weighted average of absolute color difference to enable more uniform distribution of the original image. It divides the dynamic range of the histogram into *k* equal subhistograms and assigns the dynamic range based on their area ratios. Then, DRSHE reassigns the intensities of the histogram uniformly in the resized dynamic range. DRSHE can preserve the naturalness of the original image and avoid severe change in brightness. In addition, in [5], statistic-separate tri-histogram equalization (SSTHE) uses the values *μ* − *w × σ* and *μ +* *w × σ* to separate the histogram of an image into three regions and revises the range of each region. Here, *w* is an adjustable weighting value. *μ* and *σ* are the mean and the standard deviation of the image, individually. Then, SSTHE performs HE within each region. Bi-histogram equalization with a plateau level (BHEPL) [6] uses the mean value to segment the input histogram into two subhistograms. It clips every subhistogram according to the plateau limits to avoid over-amplification of noise. Next, two transform functions proposed by the authors are applied to these two subhistograms to make them be equalized. Weighting mean-separated sub-histogram equalization (WMSHE) [7] segments the histogram based on the proposed weighting mean function. Next, WMSHE equalizes each subhistogram individually. The methods described above use various ways to divide the histogram into some subhistograms. After that, HE or other equalization methods are applied to stretch the dynamic range of an image to achieve the purpose of image enhancement. These methods can avoid the over-enhancement problem because each subhistogram is limited within a certain range; nevertheless, they cannot deal with the feature loss problem produced by HE.

Further, many studies focused on preserving the image brightness and maintaining image quality. Equal area dualistic subimage histogram equalization (DSIHE) [8] segments an image into two equal subimages by using the median value. Then, DSIHE executes HE within each subimage. The enhanced images by DSIHE can maintain the brightness of the original image. Similar to BBHE, recursive mean-separate histogram equalization (RMSHE) [9] uses the mean value to segment the input histogram into two subhistograms. RMSHE performs the separation recursively *r* times, and then, each subhistogram is equalized independently. When *r* becomes greater, the output mean is close to the input mean. Hence, RMSHE can provide better brightness preservation. Minimum mean brightness error bi-histogram equalization (MMBEBHE) [10] tries to find out the specific value from gray level 0 to *L*-1 that obtains the minimum absolute difference value of the input and output means. Then, MMBEBHE utilizes the specific value to segment the input histogram into two subhistograms and equalizes them separately. The enhanced images by MMBEBHE can preserve the maximum brightness of the original image. Brightness preserving histogram equalization with maximum entropy (BPHEME) [11] decides a histogram which has the maximum entropy. Hence, it preserves the brightness of an original image considerably well. Recursive sub-image histogram equalization (RSIHE) [12] is similar to DSIHE in its usage of the median value to separate the input histogram into two subhistograms. RSIHE performs the separation recursively *r* times, and HE is performed on each subhistogram. The average brightness of the image obtained by applying RSIHE is equal to the value obtained in DSIHE. Thus, RSIHE can achieve brightness preservation. Brightness preserving dynamic histogram equalization (BPDHE) [13] uses the concepts of MPHEBP and DHE. BPDHE utilizes the local maxima of the smoothed histogram to divide the histogram, and is thus similar to the approach used by MPHEBP. In addition, the step that BPDHE maps each segment to a new dynamic range is close to the approach utilized by DHE. Next, BPDHE normalizes the output intensity. The average intensity of the processed image is almost the same as that of the original image. Thus, BPDHE can effectively preserve the brightness of images. Flattest histogram specification with accurate brightness preservation (FHSABP) [14] determines the flattest histogram with mean brightness constraint. Then, it acquires better brightness preservation by using an exact histogram specification. A histogram modification framework for image contrast enhancement [15] adjusts the levels of enhancement by tuning the parameters of the introduced penalty terms manually. The processed images have good enhancement quality. Dynamic quadrants histogram equalization plateau limit (DQHEPL) [16] segments the histogram based on the median value and iteratively generates four subhistograms. Then, DQHEPL uses the plateau limit, the average intensity of each subhistogram, to clip each subhistogram. After that, each subhistogram is assigned to a new dynamic range. Finally, DQHEPL performs HE within each subhistogram individually. The images processed by DQHEPL can avoid over enhancement and maintain the mean brightness. Piecewise maximum entropy (PME) [17] obtains the maximum entropy by using the piecewise transformation function. The resultant images obtained after using PME preserve the original brightness considerably well. The methods described above endeavor to resolve the problem of severe change in brightness produced by HE and to maintain the brightness of the original images to achieve the purpose of image enhancement. They are proposed to maintain the brightness of the resultant image almost at the same level as that of the original. Nevertheless, when the images are overexposed or underexposed, maintaining the brightness of the input images is not logical because in such cases, the effects of enhancement would not meet the needs for human visual perception.

Apart from the abovementioned methods, the contextual and variational contrast enhancement algorithm (CVC) [18] employs contextual data modeling using two dimensional (2D) histogram of the input image to execute nonlinear data mapping for producing pleasing enhanced image. Lee *et al.* [19] also brought out a contrast enhancement algorithm using the difference of 2D histograms of gray-level differences between adjacent pixels to amplify the gray-level differences between adjacent pixels to reach the goal of image enhancement. Huang *et al.* [20] proposed an efficient contrast enhancement using adaptive gamma correction with weighting distribution (AGCWD). It improves the brightness of darkish images by means of the gamma correction and probability distribution of luminance pixels. Due to the limitation of the gamma correction, AGCWD is suitably applied to the dimmed images. Ting *et al.* [21] proposed a visual contrast enhancement algorithm (VCEA), which takes the requirements of the human visual perception into consideration and tries to alleviate the over enhancement and feature loss problems. In addition, VCEA further improves the detailed textures of an image. The resultant images have better visual quality.

This paper refers to the VCEA method and proposes a contrast enhancement algorithm based on gap adjustment for histogram equalization (CegaHE) to further improve the enhancement effects of VCEA. CegaHE also considers the properties of human visual perception. It mitigates the over enhancement and feature loss problems produced by HE. Further, CegaHE enhances the textures in the dark regions of the images and improves their clarity. Images obtained by applying CegaHE are more suitable for human visual perception and have better visual quality than those obtained by applying VCEA and other HE-based methods.

The remainder of the paper is structured as follows. The proposed algorithm, CegaHE, is introduced in Section 2. The experimental results of CegaHE and other HE-based methods are demonstrated in Section 3. Finally, the conclusions are stated in Section 4.

## 2. The Proposed Algorithm

**Figure 1:**
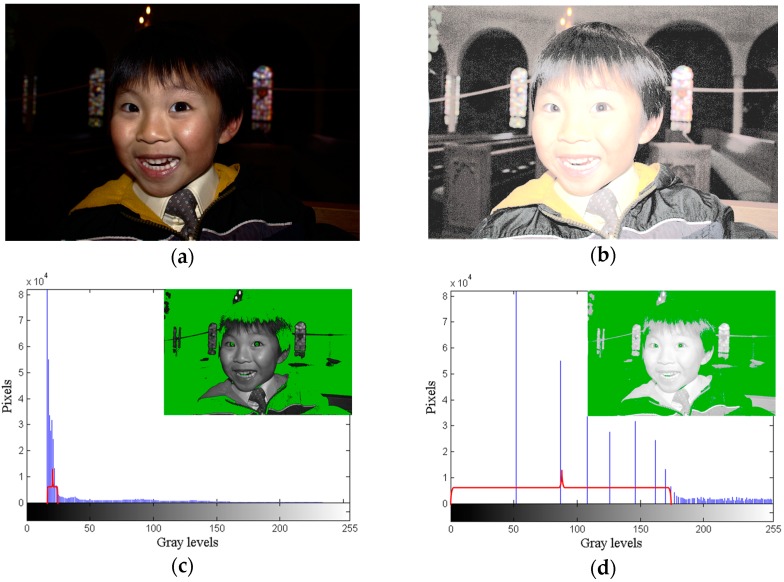
*Daniel* image (image size: 1935 × 1294 pixels). (**a**) Original image; (**b**) HE image; (**c**) original histogram; (**d**) HE histogram.

HE is a simple and effective method for improving image contrast; however, it may cause the over-enhancement and feature loss problems. The basic principle of HE is to remap the gray values of the original image to new ones based on the cumulative distribution function by stretching the dynamic range to improve the image contrast. Figure 1a,b represent the dark and low contrast original image, and the HE image, respectively. Figure 1c,d are the histograms of the Y component of Figure 1a,b individually. Figure 1b illustrates the over-enhancement problem, in which the face and clothing of Daniel, as well as the background of the image are very bright and look unnatural. The reason for this problem is that HE stretches the gaps of the histogram between two adjacent gray values to a great extent as shown in Figure 1d, thus leading to over enhancement. In this paper, the term “gap” denotes the number of gray levels between two neighboring gray values. Further, HE also causes the feature loss problem. HE expands the dynamic range of the original image from gray level 0 to 255 according to the cumulative probability of each gray value. Hence, when the probability of a particular gray value is less than 1/256, it cannot be distributed a gray level. It is combined with other gray values until the cumulative probability is greater than 1/256 and can be distributed at least one gray level. All such gray values map to the same gray value. The above process continues until no gray level remains. Many compressed gray values mapping to the same one results in the feature loss problem. In Figure 1b, the facial and clothing features of Daniel are fewer than in the original image. Further, many details cannot be seen clearly. Many researchers proposed various methods to overcome the limitations of HE. However, they focused on methods to solve the over-enhancement problem, and did not consider the problem of gray values being compressed, which causes the enhanced images to lose some features. Further discussions related to the small images in Figure 1c,d are presented in Section 2.1.

**Figure 2:**
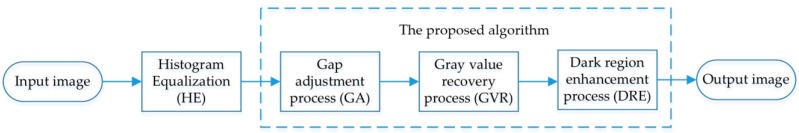
The block diagram of CegaHE.

The proposed algorithm takes the needs of human visual perception into consideration and alleviates the over enhancement and feature loss problems caused by HE. Further, CegaHE can enhance the textures in the dark regions of the images and make them clearer. Figure 2 shows the block diagram of CegaHE.

CegaHE is a HE-based method. Therefore, all the input images processed by CegaHE need to be processed by HE in advance. After that, they are processed by each process of CegaHE. The three major processes in CegaHE are as follows:

- Gap adjustment process (GA)
  This process adjusts the gaps between two neighboring gray values in the HE histogram to alleviate the over-enhancement problem produced by HE, and generates processed images that meet the minimum requirement of human visual perception. Here, the minimum requirement of human visual perception indicates that when observing the image carefully, human visual perception can discern the differences between features of the image without difficulty. After the adjustment, the “free pixels”, *i.e.*, the available gaps, are used in the following two processes:
- Gray value recovery process (GVR)
  This process alleviates the feature loss problem produced by HE. HE merges the gray values with low cumulative probabilities to the same gray value, resulting in the feature loss problem. To mitigate the problem, the GVR uses free pixels to recover the lost features.
- Dark region enhancement process (DRE)
  Owing to the inferior light sensitivity of CCD/CMOS sensors, the image quality is poor, especially when the light is dim or insufficient. To compensate for this effect, the process enhances the textures in the dark regions of the images, and increases their contrast and clarity.

In this paper, CegaHE adopts the RGB color space to process the input images. Prior to applying CegaHE, the RGB color space is converted to the YUV color space. YUV consists of one luminance (Y) component and two chrominance components (U and V). For color images, the luminance (Y) component is used for further processing. For gray images, CegaHE utilizes gray values for further processing. Then, CegaHE uses the HE histogram of an image as the basis to adjust the gaps between two neighboring gray values to meet the minimum requirement of human visual perception. HE can be used to stretch the intensity histogram of an image and obtain better contrast. However, HE may widen the gaps of gray values excessively, and cause the over-enhancement problem. In order to solve this problem, the GA refers to the characteristics of human visual perception and adjusts the gaps of neighboring gray values using a proposed adjustment method to restrict the overstretched situation. The details of the GA are illustrated in Section 2.1.

### 2.1. Gap Adjustment Process (GA)

The GA alleviates the problem of overstretched gaps produced by HE and ensures that the resultant image meets the minimum requirement of human visual perception. In Figure 1a, the face and clothing of Daniel can be observed, but the background details are not clearly seen. In Figure 1c, the green regions, which occupy two-thirds of the total area of the small image, have seven gray values that lie in the dark region of the dynamic range. Therefore, the details of the background are not easily seen. On the contrary, other parts of the small image with 211 gray values, which are spread from gray level 23 to 233, occupy most of the dynamic range, and therefore, most facial and clothing features of Daniel can be observed clearly. In Figure 1b, the face and clothing of Daniel are too bright to be seen after the process of HE. According to the cumulative distribution function of an image, HE flattens the dynamic range from gray level 0 to 255. That causes the gray values with greater cumulative probabilities to be spread extensively, resulting in over enhancement. From Figure 1d, the green parts in the small image have seven gray values that occupy approximately two-thirds of the dynamic range in the red over brace. Seventy-six other gray values, which are compressed from the original 211 gray values, shift to the bright region of the dynamic range and occupy one-third of the dynamic range. Thus, there is an information loss corresponding to 135 gray values. Therefore, in comparison with the original image, Figure 1b loses some features and also exhibits the over-enhancement problem.

In general, human visual perception is more sensitive to luminance change in the middle of the luminance range [22,23,24]. Conversely, it is less sensitive to luminance change in the dark or bright regions of the luminance range. Thus, human visual perception can discern only a narrow gap in the middle of the luminance range but a wide gap in the bright and dark areas of the luminance range. HE stretches the gaps between two neighboring gray values excessively owing to the greater cumulative probabilities, thus resulting in over-enhancement. Therefore, the gaps between two adjacent gray values should be adjusted adequately to avoid over-enhancement. According to the properties of human visual perception and the concept of adjusting gaps, the proposed adjustment equation, the gap limiter, is used as the basis to restrict the gaps between gray values. The proposed gap adjustment is more consistent with human visual perception. When the gaps between two neighboring gray values are greater than the corresponding values of the gap limiter, the gaps should be narrowed based on the values of the limiter in order to make the processed image meet the minimum requirement of human visual perception. The gap limiter equation is:

(1) $$L(G)=Round\left(a\times {\left[\left(G/127\right)-1\right]}^{2}\right)+b$$

where *L*(*G*) denotes the limiting gap of each gray value, *G* is the gray value ranging from 0 to 255, and *a* and *b* are parameters that control the levels of enhancement. *Round* denotes to round *a* × [(*G*/127) – 1]^2^ to the nearest integer. When *a* and *b* increase, the gaps between gray values also increase, and the features of the adjusted image become clearer. However, this would reduce the number of free pixels remaining for the succeeding processes to use. When the gap between two neighboring gray values is greater than the value of the gap limiter calculated by using Equation (1), the difference between the gap and the gap limiter is defined as “free pixels”.

**Figure 3:**
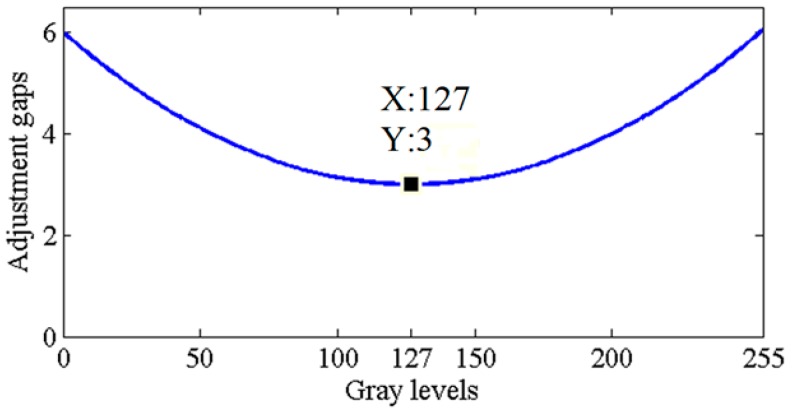
Gap limiter.

The task of selecting values of *a* and *b* that help to obtain better images and more free pixels is a tradeoff challenge. The objective is to generate GA images that have as many features as possible to meet the minimum requirement of human visual perception, and to simultaneously acquire more free pixels for the succeeding processes. In order to obtain adequate adjustment gaps, the GA processes different types of images such as underexposed, overexposed, low contrast, and high contrast images to obtain suitable *a* and *b*. From the processed images, we deduce that when both *a* and *b* are equal to 3, the processed images can meet the minimum requirement of human visual perception, and more free pixels can be obtained. The choice of values of *a* and *b* is discussed further in the Appendix A. Figure 3 shows the adjustment curve of the gap limiter for each gray value.

**Figure 4:**
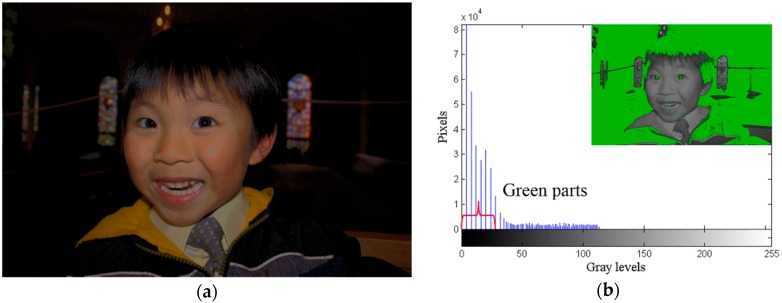
*Daniel* image, (**a**) GA image; (**b**) GA histogram.

After the image is processed by HE, suppose *his*(*x*) denotes the number of pixels at gray value *x*, ranging from 0 to 255, and the gap between gray value *x* and the one previous *x* is *d*. If *d* is more than the value of the gap limiter, *L*(*x*), *his*(*x*) is moved backward by *d* − *L*(*x*) gray levels. After all *his*(*x*) are moved backward, the GA image is obtained as shown in Figure 4a.

In Figure 4a, the details of the face and clothing of Daniel can be seen. At the same time, human visual perception can perceive the differences between the above features of the image without difficulty. Furthermore, in Figure 1b, the face and clothing of Daniel consist of 76 gray values ranging from gray level 176 to 255, which occupy the bright region of the dynamic range. After the GA, those features have the same number of gray values ranging from gray level 31 to 110, which move to the middle of the dynamic range in Figure 4b. Human visual perception has good discrimination in the middle region of the dynamic range, and therefore, Figure 4a has a better visual effect than Figure 1b. Further, the dynamic range of the green regions, which occupy two-thirds of the area of the small image in Figure 4b with seven gray values, is wider than the dynamic range of the green regions of the small image in Figure 1c. Therefore, the details of the background of Figure 4a are clearer than those of the background of Figure 1a. Thus, the GA solves the over-enhancement problem, retains the characteristic of HE that enhances image contrast, and meets the minimum requirement of human visual perception. The obtained 145 free pixels are used by the succeeding processes for further enhancement.

### 2.2. Gray Value Recovery Process (GVR)

HE and other HE-based methods proposed until now result in the feature loss problem. They stretch the gaps between two adjacent gray values depending on cumulative probabilities. When cumulative probabilities of gray values are not high enough to be allocated a gray level, they are combined to a certain gray value whose cumulative probability is high enough to be allocated at least one gray level. This phenomenon of many gray values being merged to the same one causes the feature loss problem. In Figure 1a, the number of gray values of the *Daniel* image is 218. After performing HE, the number of gray values becomes 83. Thus, 135 gray values of the features are compressed. Therefore, this paper proposes the GVR to alleviate the feature loss problem.

The GVR recovers the lost gray values of an image by using the free pixels that are available after the GA. The recovery of the features counts on the number of free pixels. When the number of free pixels is greater than or equal to twice that of the compressed gray values, the GVR recovers all the lost features sequentially. However, when the number of free pixels is less than twice that of the compressed gray levels, the GVR recovers the features with pixels greater than the value, *image size* × (1/255) × *β*, sequentially. When *β* increases, fewer features can be recovered; conversely, when *β* decreases, more features can be recovered. After experimenting with different values of *β*, we observe that the GVR can recover more features for the majority of the images when the value of *β* is 0.1. After the GVR, the processed image recovers most features of the original image.

**Figure 5:**
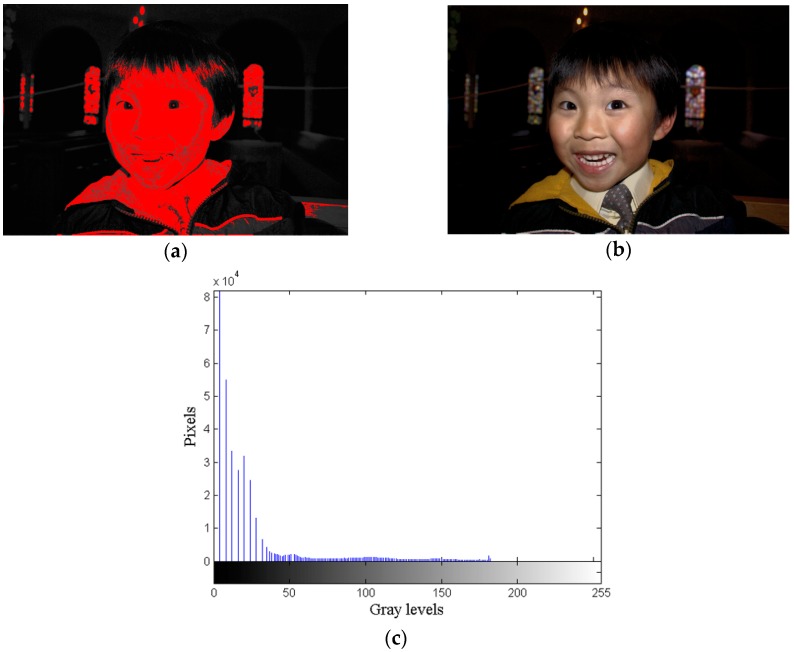
GVR results for *Daniel* image, (**a**) Regions of compressed gray values; (**b**) GVR image; (**c**) GVR histogram.

Although Figure 4a can meet the minimum requirement of human visual perception and solve the over-enhancement problem of HE, the feature loss problem of HE still exists. Hence, features such as the color gradient of facial complexion, the shirt, and the tie have disappeared, and the quality of the image has degraded. The red regions in Figure 5a are the regions where features are lost caused by HE. After performing the GVR, 68 compressed gray values are recovered as shown in Figure 5b, and 77 free pixels remain for use by DRE. Figure 5b looks better, more vivid, and clearer than Figure 4a. This proves that the GVR can effectively alleviate the feature loss problem.

### 2.3. Dark Region Enhancement Process (DRE)

The DRE enhances the textures in the dark region of an image and compensates for the disadvantage of inferior light sensitivity of CCD/CMOS sensors. When taking pictures, the images are generally extremely dark owing to insufficient light or low light sensitivity of CCD/CMOS sensors. Further, the textures in the dark regions of the images are not easily discerned by human visual perception. Therefore, the DRE focuses on these textures for further enhancement.

**Figure 6:**
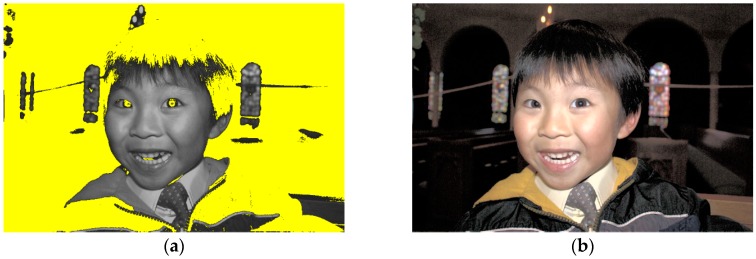

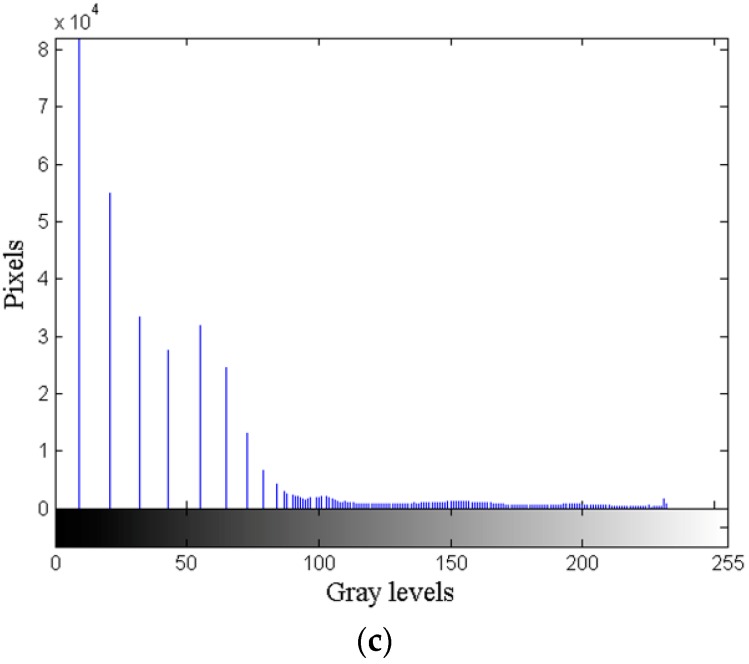
DRE results for *Daniel* image, (**a**) Dark region of the GVR image; (**b**) DRE image; (**c**) DRE histogram.

First, the DRE segments the GVR image into the dark and the bright regions by using its mean. Pixel values less than the mean value imply that the pixels lie in the dark region. The yellow area of Figure 6a shows the dark region of the *Daniel* image. Then, the DRE computes the gradient magnitude at each pixel and sums up the gradient values of the gray values in the dark region of the image.

For example, $n_{i,j}$ represents the value of each pixel in an image and the size of the image is W×H pixels. The gradient value $G_{n_{i,j}}$ of $n_{i,j}$ is equal to the sum of $\left|GH_{n_{i,j}}\right|$ and $\left|GV_{n_{i,j}}\right|$. $\left|GH_{n_{i,j}}\right|$ and $\left|GV_{n_{i,j}}\right|$ are equal to $\left|n_{i,j+1}-n_{i,j-1}\right|$ and $\left|n_{i+1,j}-n_{i-1,j}\right|$, respectively:

(2) $$G_{n_{i,j}}=\left|GH_{n_{i,j}}\right|+\left|GV_{n_{i,j}}\right|=\left|n_{i,j+1}-n_{i,j-1}\right|+\left|n_{i+1,j}-n_{i-1,j}\right|\;$$

where 0≤i≤W−1 and 0≤j≤H−1. Suppose *G*(*x*) is the gradient sum of the *x*th gray value of the dark region. The DRE computes the gradient probabilities, P(x), of the gray values of the dark region:

(3) $$P(x)=G(x)/\sum_{r=0}^{mean-1}G(r)\;0\leq x\leq mean-1\;$$

where *mean* is defined as:

(4) $$mean=\sum_{s=0}^{255}s\times p(s)\;$$

where *p*(*s*) is the ratio of the total pixels of each gray level to the total pixels of the whole image. Further, the DRE adopts the remaining free pixels, *rfp*, to enhance the textures in the dark region. In order to avoid over-enhancement, the DRE utilizes only a part of the remaining free pixels, *R_rfp_*, based on the ratio of the number of pixels in the dark region to the total number of pixels. Suppose *D*(*x*) is the total number of pixels at gray value *x*. The *R_rfp_* is defined as:

(5) $$R_{rfp}=\left[\left(\sum_{x=0}^{mean-1}D(x)/\sum_{x=0}^{255}D(x)\right)\right]\;\times rfp\;$$

Based on the gradient probabilities of each gray value in the dark region, the DRE distributes *R_rfp_* to enhance these gray values. The allocated gray level, *Space*(*x*), of the *x*th gray value is defined as:

(6) $$Space(x)=R_{rfp}\times P(x)\;0\leq x\leq mean-1\;$$

Finally, the DRE expands the gaps of the gray values in the dark region based on *Space*(*x*). The image processed by the DRE is shown in Figure 6b. The DRE uses 54 free pixels, and 23 free pixels remain. Owing to the expansion of the gaps of the gray values in the dark region, features such as the contours of tables, benches, and arches behind Daniel can also be manifested. The background of Figure 6b becomes clearer than the one of Figure 5b, the GVR image. Thus, it is seen that the DRE can effectively enhance the textures in the dark region of an image.

## 3. Experimental Results

**Figure 7:**
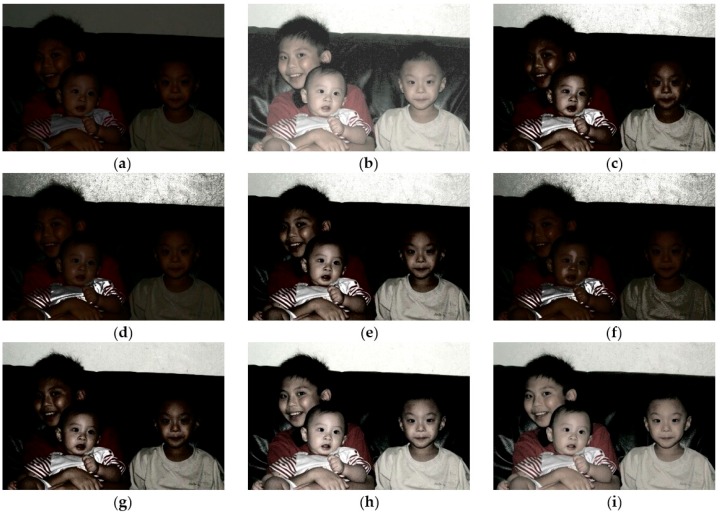
Results of different methods for the image *Brothers* (image size: 960 × 640 pixels): (**a**) Original image; (**b**) HE; (**c**) BBHE; (**d**) RMSHE (r = 2); (**e**) DSIHE; (**f**) RSIHE (r = 2); (**g**) DQHEPL; (**h**) VCEA; and (**i**) CegaHE.

**Figure 8:**
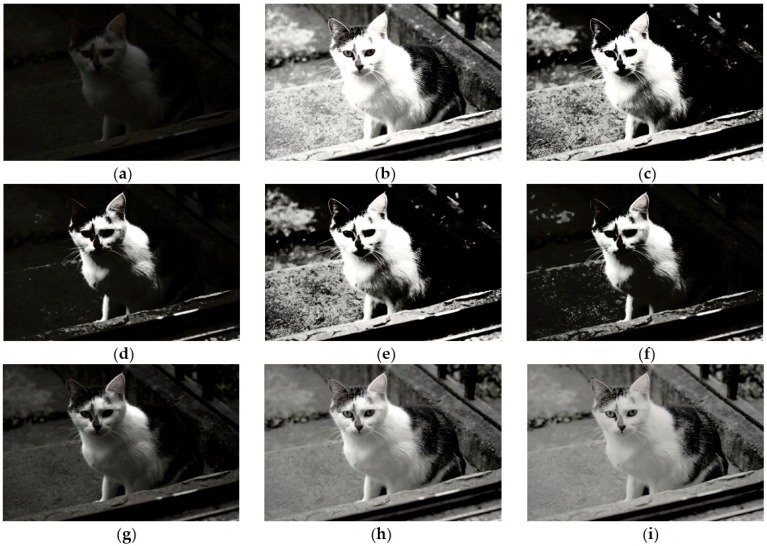
Results of different methods for the image *Cat* [26] (image size: 902 × 600 pixels): (**a**) Original image; (**b**) HE; (**c**) BBHE; (**d**) RMSHE (r = 2); (**e**) DSIHE; (**f**) RSIHE (r = 2); (**g**) DQHEPL; (**h**) VCEA; and (**i**) CegaHE.

**Figure 9:**
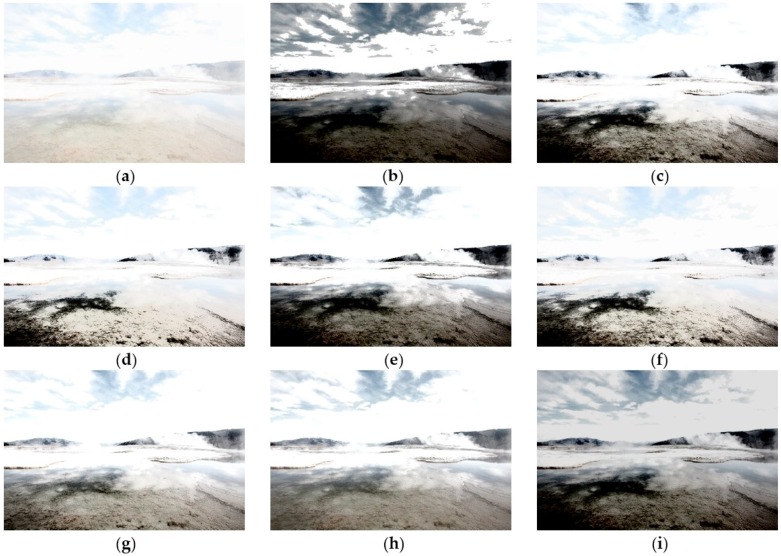
Results of different methods for the image *Landscape* [27] (image size: 960 × 640 pixels): (**a**) Original image; (**b**) HE; (**c**) BBHE; (**d**) RMSHE (r = 2); (**e**) DSIHE; (**f**) RSIHE (r = 2); (**g**) DQHEPL; (**h**) VCEA; and (**i**) CegaHE.

**Figure 10:**
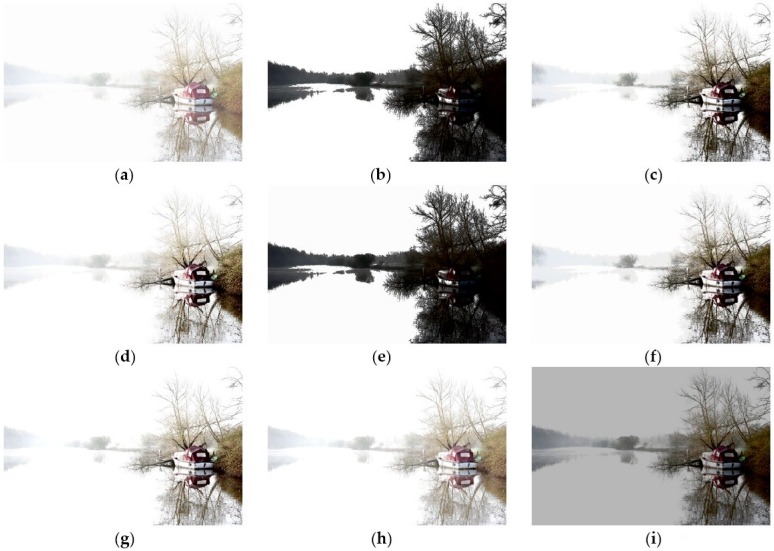
Results of different methods for the image *Boat* [28] (image size: 900 × 600 pixels): (**a**) Original image; (**b**) HE; (**c**) BBHE; (**d**) RMSHE (r = 2); (**e**) DSIHE; (**f**) RSIHE (r = 2); (**g**) DQHEPL; (**h**) VCEA; and (**i**) CegaHE.

To illustrate the preeminence of CegaHE, both subjective and objective assessments are utilized in this paper to compare the results of CegaHE with the ones of other well-known methods. As far as subjective assessment is concerned, Figure 7, Figure 8, Figure 9 and Figure 10 show the experimental results of CegaHE and other methods, namely, HE, BBHE [2], RMSHE [9], DSIHE [8], RSIHE [12], DQHEPL [16], and VCEA [21]. In terms of objective assessment, the discrete entropy (E) [25] is used to measure the capability of extracting details from images. A higher discrete entropy denotes that more information is extracted from an image. The detailed statements concerning subjective and objective assessments are as follows.

### 3.1. Subjective Assessment

Figure 7a is a standard image with underexposure. Figure 7b, processed by HE, is over- enhanced and produces unpleasant visual artifacts. The background noise is also amplified by HE. Figure 7c–g are the results after applying BBHE, RMSHE, DSIHE, RSIHE, and DQHEPL, individually. They are exceedingly dark and the faces of the brothers and the textures of the sofa are not seen clearly.

Figure 7h processed by VCEA shows the faces and the clothing of the brothers as well as the textures of the sofa in the dark region of the image clearly. However, some parts of the image are brighter, e.g., the faces of the brothers and the wall behind the sofa. Figure 7i, the result after applying CegaHE, shows not only the faces and the clothing of the brothers but also the textures of the sofa in the dark region of the image clearly. It looks more natural and has better visual quality than the images processed by other methods.

Figure 8a is an underexposed image. The detailed body textures of the cat and the background are not seen clearly. Figure 8b, the result of processing by HE is an over enhanced image in which some textures of the cat are not seen. Figure 8c–f are processed by BBHE, RMSHE, DSIHE, and RSIHE, individually. The problem they exhibit is that the dark regions appear extremely dark and the bright regions appear extremely bright. The images obtained using these methods appear unclear and unnatural. Figure 8g, processed by DQHEPL, is much better than the ones obtained using the other methods. However, details of the left eye are not clear and some detailed body textures of the cat appear too dark to be seen clearly. Figure 8h, the image obtained by VCEA, has good enhancement performance. All the details can be seen clearly. However, the forehead, the body, and the right front foot of the cat are too bright. Figure 8i, the result of processing using CegaHE has the best enhancement performance. The detailed body textures of the cat are recovered; hence, the features and color gradient of the body of the cat are seen clearly. Further, owing to the enhancement of the dark textures of the image, the details of the background and the left eye of the cat can be seen clearly. Thus, Figure 8i looks more natural and has better visual quality.

Figure 9a is a low contrast and high brightness image. Figure 9b–h are obtained after processing the image using HE, BBHE, RMSHE, DSIHE, RSIHE, DQHEPL, and VCEA, respectively. All these resultant images exhibit the overexposure problem, with the sky and the ice layers in the images being extremely bright, thus making them appear unnatural. The surface of Figure 9b is extremely dark, and therefore, some textures not easily visible. The surface in Figure 9d,f is extremely bright, causing some features of the surface to disappear. Figure 9i, obtained by applying CegaHE, is clearer than the images obtained by using the other methods. The textures of the sky, the surface, and the ice layers are clear. In addition to higher contrast, the resulting image has better visual quality.

Figure 10a is a low contrast and high brightness image. Figure 10b,e are obtained by applying HE and DSIHE, individually. The problem of Figure 10b,e is that it is hard to see the boat, trees, and their reflections clearly because they are too dark. Figure 10c,d,f–h also have the same problem with the trees and their reflections in the distance being too bright to be seen clearly. Figure 10i, which is processed by CegaHE, has better visual quality. The trees and their reflections in the distance are much clearer. Further, the detailed textures of the boat, trees behind the boat, and their reflections on the lake are recovered by CegaHE, and displayed clearly. In addition, the resulting image appears more natural than the ones obtained by applying the other methods.

The remaining free pixels of each image after each process are recorded in Table 1. From Table 1, we can observe that free pixels remain for each image. These free pixels can be used for further contrast enhancement.

Thus, CegaHE alleviates the limitations of HE, *i.e.*, the over enhancement and feature loss problems, and also improves the clarity of the textures in the dark regions of the images. In the case of underexposed images, CegaHE improves their clarity, resulting in large amount of detail and makes them adequate for human visual perception. In the case of overexposed images, CegaHE effectively restricts the over-enhancement effect and makes the resultant images appear more natural. Comparing with VCEA and other HE-based methods, CegaHE yields better visual quality images.

### 3.2. Objective Assessment

Apart from subjective assessment of the enhancement effect by means of observation, objective assessment, which is the quantitative measure, is often used to verify the enhancement effect in most studies. It is not easy to find a suitable quantitative measure. Consequently, one of the objective assessment methods, the discrete entropy [25], is utilized in this paper to quantitatively evaluate the performance of different enhancement methods. The definition of discrete entropy *E* is as follows:

(7) $$E\;=\;-\sum_{i=0}^{j}p(X_{i})\;\times \;log_{2}p(X_{i})$$

where *j* is the maximum intensity value (e.g., for 8-bit images, *j* = 255) and *p*(X*_i_*) is the probability at gray level *i*. Table 2 shows the discrete entropy values of compared methods. The higher entropy value indicates that more information is extracted from an image. However, the images with higher entropy values do not always denote that their enhancement effects are better than others. To take an example, DQHEPL has the highest entropy values for Figure 7, Figure 8, Figure 9 and Figure 10 in Table 2. It means that DQHEPL obtains significant information from the original images. In fact, enhancement effects of DQHEPL are not as good as the ones of VCEA and CegaHE. Although objective assessment provides quantitative information to readers; nevertheless, it must be accompanied by subjective assessment. The reason is that subjective assessment is the most direct method to evaluate image quality from observers [21].

In Table 2, CegaHE shows the highest entropy for Figure 9, which is the same value as DQHEPL and VCEA, indicating that CegaHE has the capability of extracting significant information from the original image. Besides, CegaHE has the second highest entropy values in Figure 7, Figure 8 and Figure 10. All these values are almost close to the highest entropy values. That denotes that the capability to extract information of CegaHE is almost the same as the ones of DQHEPL and VCEA. Although the entropy values of CegaHE are not always the highest, subjective assessment shows that CegaHE has better enhancement effect than other methods in Figure 7, Figure 8, Figure 9 and Figure 10.

The purpose of image enhancement is to improve the quality of an image and to make the enhanced image suitable for human visual perception. Therefore, both subjective and objective assessments are often used by most researchers to compare their results with the ones of other methods. Few studies compare the execution times of different image enhancement methods especially for an image because most studies focus on the image quality instead of its execution time for an image. However, for continuous images such as video images, because they have to be processed in real time, the execution time needs to be taken into consideration. According to the standard set up by NTSC, the frame rate is 30 frames per second. Therefore, it is high demand to have the fast execution speed to process all the video images. Currently, the operation system of the computer is 64 bit Windows 7 and the processor is Intel core i7-4790 with 12 GB RAM (Genuine, Taipei, Taiwan). Under the condition that the program of the proposed algorithm has not been optimized, the execution time of the proposed algorithm for an image of size 600 × 400 is around 70 ms. After optimizing the algorithm, the execution time can be shortened. Although the current execution speed of the proposed algorithm cannot meet the demand of the standard set up by NTSC, the proposed algorithm can provide better image quality for an image. In the future, the proposed algorithm can be optimized and applied to video images to get better image quality in real time.

To sum up, through subjective assessment described in Section 3.1, the images processed by CegaHE have better image quality and enhancement effect than the ones obtained by the other methods. Furthermore, through objective assessment, the discrete entropy is used to demonstrate the capability of extracting details from images. From Table 2, the fact that the entropy values of CegaHE are close to the highest ones shows that CegaHE has good capability of extracting details from images. All in all, both the subjective and objective analyses show that CegaHE has better enhancement effect and is superior to other methods.

## 4. Conclusions

In this paper CegaHE, a new image enhancement method, is presented. CegaHE solves the problems of HE including the over-enhancement and feature loss problems. Further, to compensate for the disadvantage of inferior sensitivity of CCD/CMOS, CegaHE enhances the textures in the dark region of an image to make them clearer. Different from VCEA, CegaHE is more consistent with human visual perception. The images processed by CegaHE are suitable for human visual perception and have better visual quality. In addition, some free pixels remain after applying CegaHE and these can be used for further contrast enhancement. The experimental results indicated that CegaHE produces better results in enhancing the contrast of the images and outperforms VCEA and other HE-based methods.

## Figures and Tables

**Table 1:** Remaining free pixels after each process.

Image	Free Pixels after the GA	Free Pixels after the GVR	Free Pixels after the DRE
Brothers	90	86	40
Cat	107	76	43
Landscape	88	82	46
Boat	147	90	82

**Table 2:** Discrete entropy values of compared methods.

Method	Brothers (Figure 7)	Cat (Figure 8)	Landscape (Figure 9)	Boat (Figure 10)
HE	4.79	4.99	4.86	3.43
BBHE	4.70	4.99	4.87	3.50
RMSHE	4.69	4.92	4.80	3.57
DSIHE	4.76	5.02	4.87	3.41
RSIHE	4.71	4.97	4.58	3.31
DQHEPL	4.82	5.05	4.88	3.64
VCEA	4.81	5.05	4.88	3.64
CegaHE	4.81	5.04	4.88	3.63

**Figure A1:**
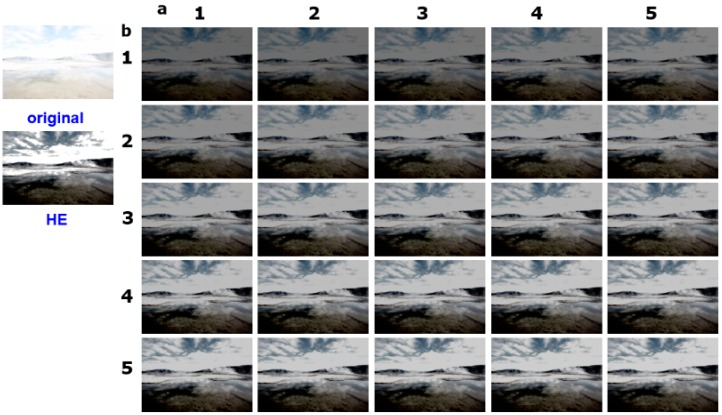
Image, *Landscape* [27], processed by GA with different *a* and *b*.

**Figure A2:**
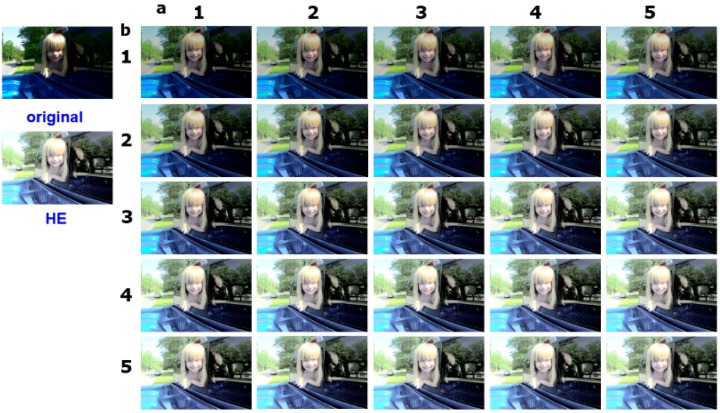
Image, *Girl* [29], processed by GA with different *a* and *b*.

**Figure A3:**
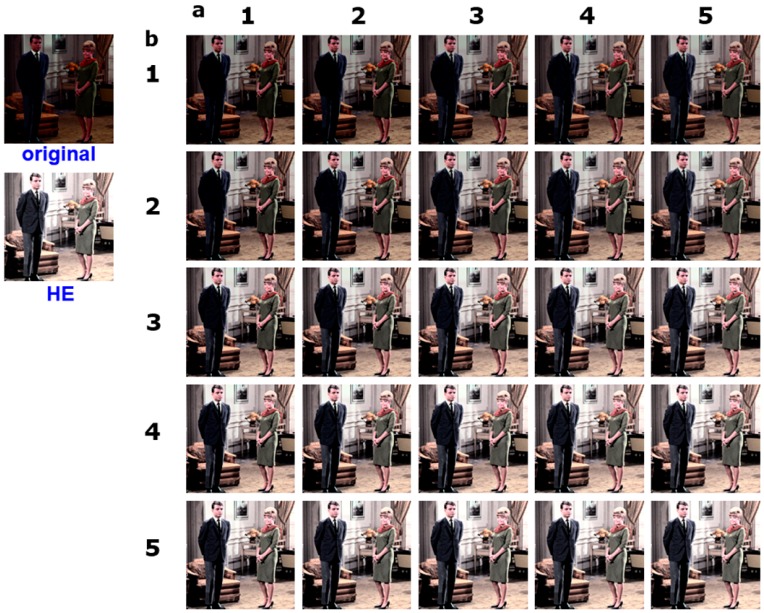
Image, *Couple* [30], processed by GA with different *a* and *b*.

## Abbreviations

The following abbreviations are used in this paper:

CCD/CMOS	Charge-Coupled Device/Complementary Metal-Oxide Semiconductor
CegaHE	Contrast Enhancement Algorithm Based on Gap Adjustment for Histogram Equalization
DRE	Dark Region Enhancement
GA	Gap Adjustment
GVR	Gray Value Recovery
VCEA	Visual Contrast Enhancement Algorithm Based on Histogram Equalization

## Appendix A

In general, human visual perception is more sensitive to luminance change in the middle of the luminance range; conversely, it is less sensitive to luminance change in the dark or bright regions of the luminance range. Based on this property of human visual perception, the GA proposes the gap limiter equation as the adjustment basis. The gap limiter equation shown in Equation (1) is where *G* is the gray value ranging from 0 to 255 and *L*(*G*) denotes the limiting gap of each gray value. *Round* denotes to round $a\;\times \;{\left[\left(G/127\right)-1\right]}^{2}$ to the nearest integer. When the gaps between two neighboring gray values are greater than the corresponding values of the gap limiter, *L(G)*, the gaps must be shortened based on *L(G)* for the processed image to meet the minimum requirement of human visual perception. In this case, the minimum requirement of human visual perception indicates that when observing the image processed by GA carefully, human visual perception can discern the differences between features of the image without difficulty. The GA reduces the over-enhancement problem caused by HE by restricting the gaps between two neighboring gray values. Simultaneously, the processed images also meet the minimum requirement of human visual perception. In order to obtain reasonable adjustment gaps, the GA processes different types of images such as underexposed, overexposed, low contrast, and high contrast images to obtain suitable values of parameters *a* and *b*. Figure A1, Figure A2 and Figure A3 show the images obtained after processing by the GA with values of *a* and *b* ranging from 1 to 5. The resultant images vary for different values of *a* and *b*. For a fixed value of *a*, as *b* increases, the GA images are brighter and more features can be seen. For a fixed value of *b*, as *a* increases, the GA images become brighter and more features can be seen. In both cases, choosing suitable values of *a* and *b* to obtain better GA images and more free pixels is a tradeoff challenge. The GA should yield images with as many features as possible and make free pixels available. In Figure A1, Figure A2 and Figure A3, we can observe that as the values of *a* and *b* increase, detailed textures and features of images can be seen more clearly. However, the number of free pixels reduces. After the GA, the resulting image need not be seen very clearly. It is important to have more free pixels and the detailed textures and features of the image should meet the minimum requirement of human visual perception. Through empirical analyses, we deduce that when the values of both *a* and *b* are equal to 3, the detailed textures and features of the GA images can be seen, and more free pixels can be obtained for the succeeding processes to further enhance images.
